# Characteristics of patients with non-severe infections of different SARS-CoV-2 omicron subvariants in China

**DOI:** 10.3389/fmed.2024.1511227

**Published:** 2024-12-18

**Authors:** Wenfang Yuan, Yongmei Liu, Haoting Zhan, Feng Wei, Qian Zhang, Huixia Gao, Huimin Yan, Tao Huang, Yongzhe Li, Erhei Dai

**Affiliations:** ^1^Division of Liver Diseases, The Fifth Hospital of Shijiazhuang, Hebei Medical University, Shijiazhuang, Hebei, China; ^2^Department of Clinical Laboratory, State Key Laboratory of Complex, Severe and Rare Diseases, Peking Union Medical College Hospital, Peking Union Medical College and Chinese Academy of Medical Sciences, Beijing, China; ^3^Hebei Key Laboratory of Immune Mechanism of Major Infectious Diseases and New Technology of Diagnosis and Treatment, The Fifth Hospital of Shijiazhuang, Shijiazhuang, Hebei, China

**Keywords:** omicron subvariant, BA.2.76, BA.5.1, clinical features, Azvudine, nucleic acid negativization, pneumonia

## Abstract

**Objective:**

The aim of this study was to explore the clinical characteristics of patients infected with different Omicron subvariants presenting non-severe disease, evaluate the safety and efficacy of Azvudine for treatment of COVID-19, in order to broaden understanding of Omicron subvariant infections.

**Method:**

A total of 244 individuals with Omicron subvariant (BA.2.76, *n* = 158; BA.5.1, *n* = 86) were included in the study. Demographic, clinical, and laboratory data of the study participants were collected and analyzed.

**Result:**

Patients infected with BA.5.1 exhibited a higher incidence of clinical symptoms like fatigue (25.58% vs. 2.53%, *p* < 0.001), headache/dizziness (12.79% vs. 4.43%, *p* = 0.017), nausea/vomiting (10.47% vs. 1.27%, *p* = 0.002), viral loads and inflammatory factors, and shorter virus shedding time than those with BA.2.76. There are 28.1% patients reporting mild adverse events following Azvudine administration. After treatment, the levels of anti-SARS-CoV-2 IgG/IgM, white blood cell, and lymphocyte obviously increased, while C-reactive protein, procalcitonin, and D-dimer reduced. Azvudine speeded up the time for virus clearance compared to control treatment (10 vs. 11 days, *p* = 0.032). Low lymphocyte counts (odd ratio (OR) = 0.607, *p* = 0.001) and anti-SARS-CoV-2 IgG titer (OR = 0.990, *p* = 0.028) were the independent risk factors for long nucleic acid negativization duration after infection. Patients with pneumonia were often accompanied by dyspnea, fatigue and high level of D-dimer. Dyspnea (OR = 10.176, *p* = 0.019) could be used to identify the occurrence of pneumonia in patients infected with Omicron.

**Conclusion:**

The study demonstrated the difference in clinical and laboratory parameters between patients infected with Omicron BA.2.76 and BA.5.1, as well as the safety and efficacy of Azvudine therapy. Our study linked patient manifestations to Omicron subvariant, treatment, and clinical outcomes, which is conducive to healthcare providers/policymakers to revise and implement appropriate countermeasures, facilitating appropriately advise for individuals with Omicron subvariant infections.

## 1 Introduction

The severe acute respiratory syndrome coronavirus 2 (SARS-CoV-2) has undergone several evolutionary changes. The incidence of infections with Variants of Concern including Alpha (B.1.1.7), Beta (B.1.351), Gamma (P.1), Delta (B.1.617.2), and Omicron (B.1.1.529) has increased worldwide (1). Compared with the other predecessors of SARS-CoV-2, Omicron showed decreased lung infectivity and became less pathogenic (2).The mortality rate of hospitalized COVID-19 patients decreased from 15.1% during Delta epidemic to 4.9% during the Omicron epidemic (3).

Furthermore, variations in symptom prevalence and treatment were observed among different Omicron subvariants (4) A prior study indicated that the hospitalization rate for febrile seizure in children was significantly higher during BA.5 predominance than during BA.1/BA.2 (5). Notably, BA.1 patients required glucocorticoids for COVID-19-associated hyperinflammatory syndrome, whereas BA.2 patients did not (6). This disparity may be attributed to the weaker induction of IFN-*β* secretion by BA.1 and BA.5 sub-lineages compared to BA.2, resulting in delayed antiviral signaling activation (7).

Additionally, the BF.7.14 and BA.5.2.48 subvariants shared identical mutation sites in the spike protein, except for R346T and C1243F (8). No significant differences in clinical manifestations, duration of illness, healthcare-seeking behaviors, or treatment were observed between these two subvariants (9). Compared with BA.2, BA.5 possesses three additional mutations: L452R, F486 V, and R493Q. Among these, R493Q enhances the infectivity of BA.5 (10). Patients infected with Omicron BA.5 exhibited faster infectivity and viral clearance as well as apparent immune escape but experienced less severe disease than those infected with BA.2 subvariant (11, 12). However, Kang’s study exhibited different results that Omicron BA.5 variant was associated with more severe and frequent systemic symptoms (13), while no difference of clinical manifestation, hospital admission, or other severe endpoints, was observed between BA.2 and BA.4/5 groups (14). Therefore, the comparison of clinical characteristics between Omicron subvariant BA.2 and BA.5 infections needs further investigation.

Azvudine could integrate RNA synthesis of SARS-COV-2 and inhibit related polymerases, ultimately terminating finally RNA replication (15). On July 25, 2022, the National Medical Products Administration approved the use of Azvudine for teh treatment of COVID-19 in adult patients, making it as the first domestic oral antiviral agent approved in China (15–17). Due to the increased transmissibility and global spread, Omicron variant has led to an outbreak in China (18). Consequently, there has been a notably increase in the number of patients seeking treatment after infection. However, there remains a scarcity of studies evaluating the effectiveness and safety of Azvudine in treating patients infected with Omicron subvariants in real-world settings.

Therefore, we conducted a retrospective study to comprehensively explore the clinical characteristics and viral kinetics between patients infected with two Omicron subvariants (BA.2.76 and BA.5.1), assessed the safety and efficacy of Azvudine, and identified the factors associated with viral clearance time and the incidence of pneumonia. This study sought to gain a broadened understanding of Omicron subvariants and provide information for designing effective treatment strategies.

## 2 Methods

### 2.1 Participant inclusion and grouping

Patients infected with Omicron subvariant BA.2.76 or BA.5.1 were retrospectively recruited into this study from the Fifth Hospital of Shijiazhuang [designated hospital for patients with Coronavirus disease 2019 (COVID-19)] between August 10, 2022, and October 9, 2022. The inclusion criteria for participants as follows: (1) aged 18 years or older; (2) positive for SARS-CoV-2 by real-time reverse transcriptase-polymerase chain reaction (RT-PCR) from nasopharyngeal and/or oropharyngeal swabs; (3) source patients of the epidemic outbreak were diagnosed with Omicron BA.2.76 or BA.5.1 infection by gene sequencing; (4) no previous infection with other SARS-CoV-2 strains from self-report and medical records; (5) was transferred to the Fifth Hospital of Shijiazhuang according to the requirements of the COVID-19 prevention and control protocol^1^ and (6) willing to participate in the study and sign the informed consent. Exclusion criteria were as follows: (1) patients with not detailed medical records and laboratory examination results; (2) patients receiving Azvudine treatment for HIV; (3) pregnant women or lactating mothers. A total of 244 patients were included in this study. We divided the patients into two groups according to the Omicron strain: BA.2.76 group (*n* = 158) and BA.5.1 group (*n* = 86).

The clinical characteristics of the enrolled patients, including COVID-19 symptoms, underlying diseases, and vaccination status, were obtained from the hospital information system at admission and collected before treatment. According to the 9^th^ edition of COVID-19 protocols for diagnosis and treatment (19), the patients recruited in the present study had non-severe (asymptomatic, mild, and moderate) illness. All participants had a clear clinical diagnosis of infection severity on the first day after admission. Nucleic acid testing was performed every one to two days for each patient during hospitalization.

Based on clinical symptoms, nucleic acid cycle threshold (CT), and lung infection, 121 patients in the study received Azvudine drug (5 mg/day for 7–14 days, Henan Genuine Biotech) and two types of self-developed Chinese medicines, including Qingre Kangdu or Lanxiang Jiedu herbal extracts (once daily). The patients in Azvudine group received Azivudine from the second (interquartile range: 1, 2) day after they tested positive for SARS-CoV-2, and the course of treatment lasted a maximum of 14 days. The non-Azvudine treatment group comprised 123 patients who only received either Qingre Kangdu or Lanxiang Jiedu herbal extracts (once daily) during hospitalization.

All patients provided informed consent, and the Institutional Review Board of the Fifth Hospital of Shijiazhuang approved this study (2022001).

### 2.2 Laboratory tests

The data on routine laboratory indices that reflect inflammation, coagulation, blood cell parameters and immunology were derived from the laboratory information system. C-reactive protein (CRP), procalcitonin (PCT), interleukin (IL), and other indexes were performed on Canon TOSHIBA-FX8 biochemistry analyzer. Blood cell counts were determined using Sysmex XN-1000 Pure Hematology Analyzer. Levels of anti-SARS-CoV-2 IgG and IgM were evaluated using YHLO iFlash 3,000 immunoassay analyzer. RT-PCR for SARS-CoV-2 was performed on Applied Biosystems 7,500 Real-Time PCR system. Except for IL, which was not assessed before discharge, all other indicators were detected on the first day of admission after infection (before treatment) and the day before discharge.

### 2.3 Statistical analysis

Statistical analysis was performed using R version 4.1.3 software, IBM SPSS Statistics version 26.0 (IBM Corp, USA), and Prism 8.0 (GraphPad, San Diego, California, USA). Normality testing was conducted using the Kolmogorov–Smirnov test. Quantitative data with a normal or non-normal distribution were expressed as mean (standard deviation, SD) and median (interquartile range, IQR), respectively. Categorical variables were presented as numbers and percentages. Independent sample t-test and Wilcoxon rank-sum test were, respectively, applied to analyze normally and non-normally distributed data. For categorical variables, the χ^2^ test was performed.

Binary logistic regression analysis was performed using SPSS software. Taking the median NAN durations of (10 days) patients in this study as threshold, binary logistic regression was initially performed to define the clinical (Azvudine treatment, BA.5.1 subvariant, fever symptom) and laboratory (lymphocyte counts and anti-SARS-CoV-2 IgG before treatment) risk factors associated with the prolonged virus clearance (>10 days) of patients infected with Omicron subvariant. The models included the adjusted Model 1 (intaking clinical parameters) and adjusted Model 2 (intaking clinical and laboratory factors).

Besides, indexes with statistical differences between patients with and without pneumonia were included in a univariable analysis, which were used to calculate odds ratios (OR) and 95% confidence intervals (CI) to explore factors associated with the incidence of pneumonia. The following factors were evaluated in the univariable analyses: fatigue (yes vs. no), dyspnea (yes vs. no), and levels of D-dimer. Moreover, statistically significant variables associated with pneumonia in the univariable analyses were further subjected to multivariable logistic regression analyses. A variance inflation factor of below 5 and tolerance of above 0.2 indicated insignificant collinearity among independent variables. Correlation analysis of the non-normally distributed data was done by Spearman’s correlation coefficients. *p* < 0.05 was considered statistically significant.

## 3 Results

### 3.1 Patients infected with subvariant BA.5.1 showed more clinical symptoms and inflammation feature

The median ages of subjects infected with Omicron subvariant BA.2.76 and BA.5.1 were 50 (35, 60.75) and 38 (34, 50) years (*p* = 0.001), respectively. Gender ratio (female to male) was similar between two groups (*p* = 0.394). Due to the higher unvaccinated rates in BA.2.76 group (8.86% vs. 1.16%, *p* = 0.022) and proved efficacy of SARS-CoV-2 vaccination on symptomatic infection (20, 21), we first analyzed the impact of vaccination status on clinical features of patients infected with BA.2.76 or BA.5.1 and observed comparable COVID-19 severity and symptoms between unvaccinated and vaccinated participants (Supplementary Table S1). Of note, patients infected with BA.5.1 had higher incidence of clinical manifestations including fatigue (25.58% vs. 2.53%, *p* < 0.001), dyspnea (5.81% vs. 0%, *p* = 0.005), abdominal pain (3.49% vs. 0%, *p* = 0.049), headache or dizziness (12.79% vs. 4.43%, *p* = 0.017), nausea or vomiting (10.47% vs. 1.27%, *p* = 0.002), and higher viral load (CT of nucleocapsid protein (CT-N): 18.16 vs. 19.82, *p* = 0.001; CT of open reading frame (CT-O): 18.14 vs. 19.43, *p* = 0.010), but shorter period for SARS-CoV-2 clearance (10 vs. 11 days, *p* = 0.028) compared with patients with BA.2.76 infection. The differences in clinical features at onset may result from the different Omicron subvariants infection.

Additionally, the absolute value of lymphocytes (*p* < 0.001), and the levels of tumor necrosis factor (TNF)-*α* (*p* = 0.024), and IL-6 (*p* = 0.026) were higher in BA.5.1 group than BA.2.76 group, while the titers of anti-SARS-CoV-2 IgG and IgM titers was comparable between the two groups at baseline (Table 1). Other laboratory indexes with no significant difference were displayed in Supplementary Table S2.

**Table 1:** Baseline characteristics of patients infected with different Omicron subvariants on admission to hospital.

Characteristics	BA.2.76	BA.5.1	*p* value
Number	158	86	**/**
Age (years)	50 (35, 60.75)	38 (34, 50)	**0.001**
Gender (F/M)	70/88	43/43	0.394
COVID-19 severity, *n* (%)			
Asymptomatic	63 (39.87%)	25 (29.07%)	0.093
Mild	78 (49.37%)	53 (61.63%)	**0.041**
Moderate	17 (10.76%)	8 (9.30%)	0.827
Symptoms, *n* (%)			
Fever	51 (32.28%)	30 (34.88%)	0.680
Fatigue	4 (2.53%)	22 (25.58%)	**<0.001**
Cough	73 (46.20%)	29 (33.72%)	0.059
Dyspnea	0 (0%)	5 (5.81%)	**0.005**
Expectoration	33 (20.89%)	19 (22.09%)	0.826
Sore throat/dry throat	43 (27.22%)	19 (22.09%)	0.380
Abdominal pain	0 (0%)	3 (3.49%)	**0.049**
Diarrhea	1 (0.63%)	2 (2.33%)	0.284
Headache/dizziness	7 (4.43%)	11 (12.79%)	**0.017**
Nausea/vomit	2 (1.27%)	9 (10.47%)	**0.002**
Myalgia	5 (3.16%)	6 (6.98%)	0.202
Vaccination status, *n* (%)			
Unvaccinated	14 (8.86%)	1 (1.16%)	**0.022**
1 dose of vaccine	1 (0.63%)	1 (1.16%)	>0.9999
2 doses of vaccine	4 (2.53%)	3 (3.49%)	0.7015
3 doses of vaccine	139 (87.97%)	81 (94.19%)	0.5105
Time after last vaccine (days)	281 (240, 388)	364 (317.5, 380.5)	**0.004**
Underlying disease, *n* (%)	40 (25.32%)	26 (30.23%)	0.409
Hypertension	27 (17.09%)	7 (8.14%)	0.054
Diabetes	8 (5.06%)	4 (4.65%)	>0.999
Cardiovascular disease	8 (5.06%)	0 (0%)	0.053
Cerebrovascular disease	4 (2.53%)	1 (1.16%)	0.659
Chronic lung disease	4 (2.53%)	0 (0%)	0.300
Chronic kidney disease	1 (0.63%)	2 (2.33%)	0.284
Chronic liver disease	2 (1.27%)	3 (3.49%)	0.348
Hematopathy	2 (1.27%)	3 (3.49%)	0.348
Malignant tumor	4 (2.53%)	15 (17.44%)	**<0.001**
Laboratory characteristics			
CT-N	19.82 (17.04, 23.52)	18.155 (15.09, 21.46)	**0.001**
CT-ORF	19.43 (17.01, 22.40)	18.145 (15.99, 20.74)	**0.010**
Time for SARS-CoV-2 nucleic acid to turn negative (days)	11 (9, 14)	10 (8,11.75)	**0.028**
Anti-SARS-CoV-2 IgG (AU/mL)	12.2 (2.8, 34.56)	8.72 (2.73, 24.09)	0.315
Anti-SARS-CoV-2 IgM (AU/mL)	0.11 (0.02, 0.25)	0.07 (0.03, 0.24)	0.755
Lymphocyte (×10^9^/L)	1.32 (1.02, 1.76)	3.81 (1.49, 4.79)	**<0.001**
Interleukin-6 (pg/mL)	2.81 (1.65, 4.9)	4.16 (2, 12.83)	**0.026**
D-dimer (μg/L)	320 (190, 462.29)	426.28 (250, 482.66)	0.051

Vaccination status refers to the vaccination status of the patient before infection. CT-N, cycle threshold of nucleocapsid protein; CT-O, cycle threshold open read frame. Bold values is that *p* < 0.05.

### 3.2 Azvudine was safe and effective in the treatment of patients infected with the omicron subvariants

The safety of Azvudine was evaluated in this current study. Adverse events had occurred in 28.1% (34/121) of the patients following Azvudine administration, such as nausea (13/121, 10.74%), diarrhea (7/121, 5.76%), vomiting (3/121, 2.47%), and headache (1/121, 0.83%). Notably, no serious adverse events (AEs) were observed for the group that received Azvudine (Table 2). Additionally, AE was found to be closely related to IL -1β levels before treatment, age, and gender (Supplementary Figure S1A). Patients with AEs, or AEs of vomit exhibited higher levels of IL-1β before treatment than those without (Supplementary Figures S1B,C). Older patients were more likely to have AEs of upset upset stomach (Supplementary Figure S1D). Nausea and vomit after Azvudine administration were more common in female patients, while liver injury was the opposite (Supplementary Figures S1E–G).

**Table 2:** Summary of adverse events following taking Azvudine in patients with Omicron infection.

Adverse events	Number
None	87 (71.90%)
Nausea	13 (10.74%)
Diarrhea	7 (5.76%)
Upset stomach	5 (4.13%)
Liver injury	4 (3.31%)
Abdominal bloating	4 (3.31%)
Vomit	3 (2.47%)
Rash	2 (1.65%)
Loss of appetite	2 (1.65%)
Headache	1 (0.83%)
Constipation	1 (0.83%)
Insomnia	1 (0.83%)

Data are displayed as number (%), representing the total number of patients who had adverse reactions (adverse events related to take Azvudine).

**Figure 1:**
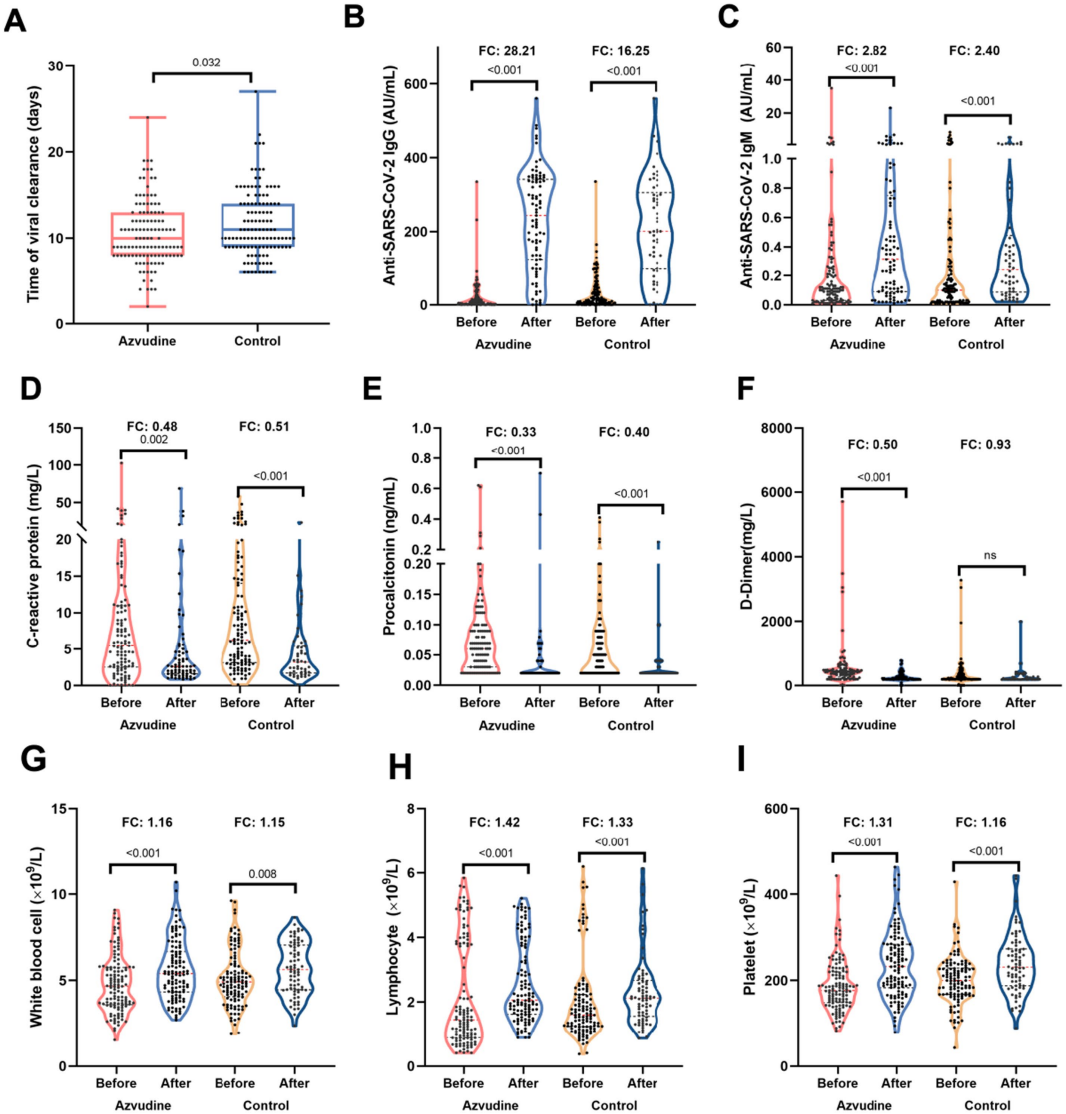
Azvudine improved the immune and inflamatory status of patients infected with Omicron variant. **(A)** The time of viral clearance was shorter in Azvudine group than control group. **(B,C)** Azvudine treatment significantly enhanced the titers of anti-SARS-CoV-2 IgG **(B)** and IgM **(C)** compared to control treatment. **(D–F)** The reduction of C-reactive protein **(D)**, procalcitonin **(E)**, and D-dimer **(F)** in azvudine group was greater than that in control group. G-H. After treatment, immune cells including white blood cell counts **(G)**, lymphocyte counts **(H)**, and platelet **(I)** of patients receiving azvudine increased more than those in control group. Statistical analysis was performed by Wilcoxon test **(A)** and paired Wilcoxon test **(B–I)**. FC, fold change.

All patients exhibited favorable prognosis, who were discharged after treatment. Patients in Azvudine group had lower proportion of asymptomatic disease (25.62% vs. 46.34%, *p* = 0.001) and higher moderate severity (18.18% vs. 2.44%, *p* < 0.001) than those in control group (Supplementary Table S3). Azvudine drug still exhibited great benefits for virus clearance, the enhancement of anti-SARS-CoV-2 antibodies and immune cells, as well as the regulation of inflammatory factors. Shorter nucleic acid negativization (NAN) durations was observed in Azvudine rather than control group (10 vs. 11 days, *p* = 0.032) (Figure 1A). The levels of anti-SARS-CoV-2 IgG and IgM, white cell counts (WBC), lymphocyte counts, and platelets (PLT) were elevated to varying degrees, whose fold changes (FC) in Azvudine group were greater than that in control group (Figures 1B,C,G–I). Meanwhile, among patients receiving Azvudine, the FC of CRP, PCT and D-dimer markedly decrease compared with patients in control group (Figures 1D–F).

Given that patients infected with BA.2.76 or BA.5.1 exhibited varying periods for SARS-COV-2 clearance (Table 1), we conducted a detailed analysis of viral clearance times, as well as alterations in anti-SARS-CoV-2 antibodies, inflammatory factors, and immune cells, specifically comparing patients who received Azvudine treatment to those in the control group within both BA.2.76 and BA.5.1 cohorts (Supplementary Figures S2, S3). Although the differences were not statistically significant, patients treated with Azvudine had shorter NAN durations compared to those in the control group, both in the BA.2.76 cohort (11.07 vs.11.83 days) and the BA.5.1 cohort (10.14 vs. 10.91 days) (Supplementary Figures S2A–S3A). In BA.5.1-infected patients who received Azvudine, there were higher increases in anti-SARSCoV-2 lgG and lgM, WBC, lymphocyte counts, PLT, and greater reductions in CRP, PCT, and D-dimer levels compared to those in the control group (Supplementary Figures S3B–I). Patients infected with BA.2.76 showed similar trends except for WBC (Supplementary Figures S2B–I).

### 3.3 Low levels of lymphocyte and anti-SARS-CoV-2 IgG before treatment prolonged SARS-CoV-2 nucleic acid negativization

**Figure 2:**
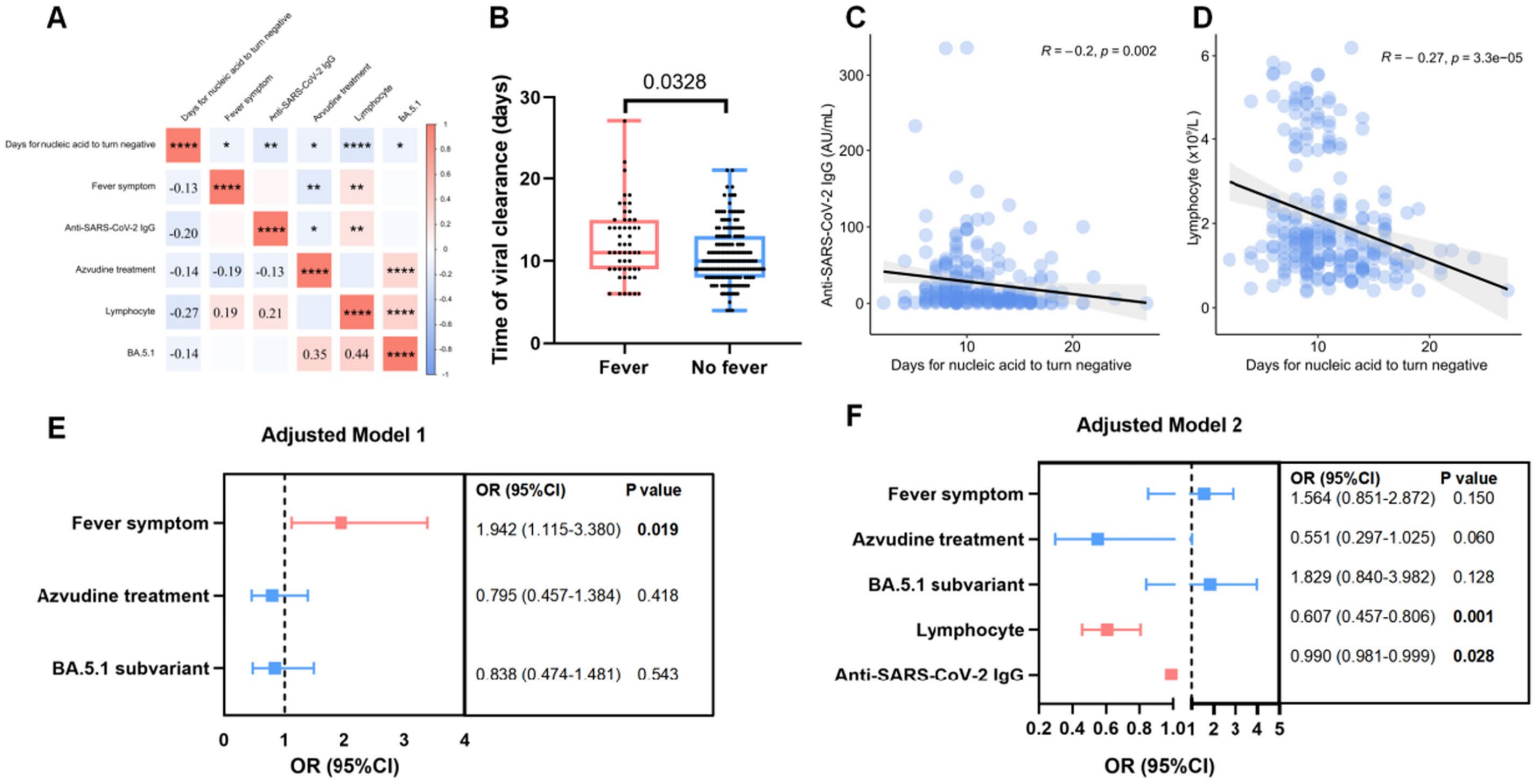
Factor associated with virus clearance in patients infected with Omicron subvariants. **(A)** The heatmap showed the factors correlated with the days for nucleic acid to turn negative after infection. **(B)** Box plot showed the difference of virul clearancr time between Omicron infected patients with or without fever symptom. **(C,D)** The association between virul clearance time (days) and lymphocytes counts **(C)** as well as anti-SARS-CoV-2 IgG **(D)**. **(E,F)** Risk factors associated with long days of nucleic acid to turn negative in model 1 **(E)** and model 2 **(F)**. Lymphocytes counts and anti-SARS-CoV-2 IgG titers were evaluated before treatment.

The correlation between the clinical and laboratory indexes and the duration of NAN was performed using Spearman’s correlation test (Figure 2A). Patients receiving Azvudine treatment (*r* = −0.14, *p* = 0.032), infected with BA.5.1 subvariant (*r* = −0.14, *p* = 0.027), without fever symptom (*r* = −0.13, *p* = 0.050) were negatively associated with SARS-CoV-2 clearance, and so is low lymphocyte counts (*r* = −0.27, *p* < 0.001) and anti-SARS-CoV-2 IgG titers (*r* = −0.20, *p* = 0.002) before treatment (Figures 1A, 2B–D). The shorter virus clearance time in the BA.5.1 group may be due to the Azvudine treatment they received.

Binary logistic regression analysis using model 1 showed that fever (OR = 1.942, 95% CI: 1.115–3.380; *p* = 0.019) was associated with >10 days NAN duration. Model 2 showed that low lymphocyte counts (OR = 0.607, 95% CI: 0.457–0.806; *p* = 0.001) and anti-SARS-CoV-2 IgG level (OR = 0.990, 95% CI: 0.990–0.999; *p* = 0.028) before treatment were independent risk factors for a prolonged NAN duration (Figures 2E–F).

### 3.4 Dyspnea was related to the incidence of pneumonia in patients infected with omicron subvariants

**Figure 3:**
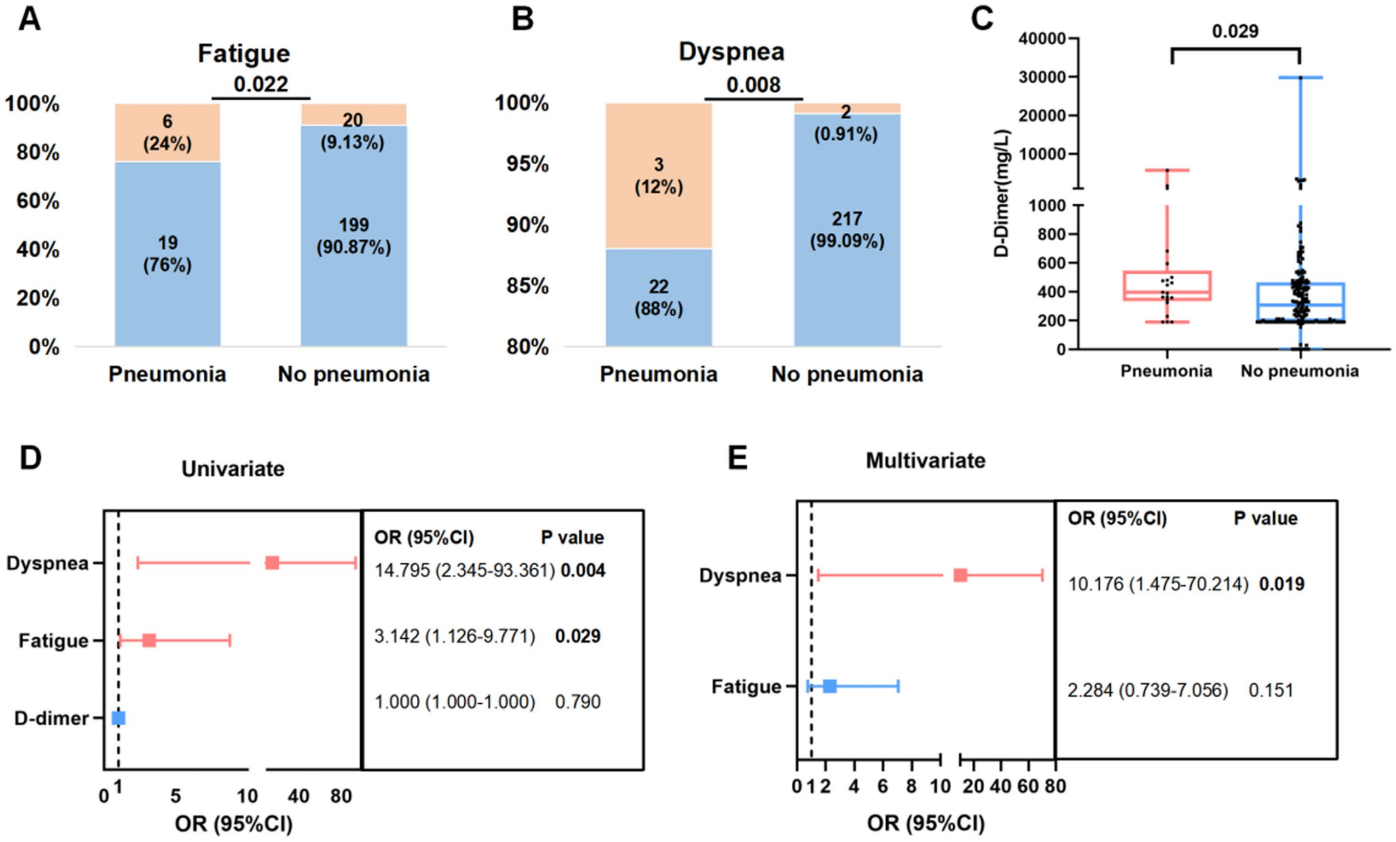
Factor associated with pneumonia in patients infected with Omicron subvariants. **(A,B)** Proportion (orange) of fatigue **(A)** and dyspnea **(B)** symptoms in Omicron infected patients with or without pneumonia. **(C)** Levels of D-dimer in patients with or without pneumonia before treatment. **(D,E)** Risk factors related to pneumonia using univariate **(D)** and multivariate logistic regression **(E)**.

In this study, 10.25% (25/244) individuals developed pneumonia. Patients with pneumonia exhibited a higher risk of dyspnea (12% vs. 0.91%, *p* = 0.008), fatigue (24% vs. 9.13%, *p* = 0.022), and a higher level of D-dimer (397 vs. 307 mg/L, *p* = 0.029) than patients without pneumonia (Figures 3A–C; Supplementary Table S3).

Then above three indicators were included in univariate logistic regression. Dyspnea (OR = 14.795, 95% CI: 2.345–93.361; *p* = 0.004) and fatigue (OR = 3.142, 95% CI: 1.126–9.771; *p* = 0.029) were associated with pneumonia. Risk factors with statistically significant differences in the univariate logistic regression were further analyzed in multivariate logistic regression, which further proved that dyspnea (OR = 10.176, 95% CI: 1.475–70.214; *p* = 0.019) was independent factors for the occurrence of pneumonia in patients with Omicron infection (Figures 3D–E).

## 4 Discussion

With the high incidence of Omicron infections but lower disease severity, Chinese authorities implemented “reopening in an orderly and effective manner” policy on December 7, 2022, before this, the city of Shijiazhuang, as a pilot area, led to large patients infected with Omicron variant. A challenge for healthcare providers and public health officials is appropriately advising individuals with different subvariants infections and implementing healthcare strategies to response Omicron pandemic. However, there is available limited information on patients infected with different Omicron subvariants. Based on this, our study (i) comprehensively exhibited the clinical and laboratory characteristics of non-severe patients infected with Omicron BA.2.76 and BA.5.1, which could help clinicians timely recognize the alterations in clinical spectrum of different subvatiants; (ii) proved the safety and efficacy of Azvudine in the treatment of non-severe COVID-19 patients to maximize their utility and guide appropriate healthcare strategies; (iii) identified the independent risk factors indicating prolonged viral clearance and pneumonia so healthcare providers can give appropriate treatment at an early stage.

Patients infected with the BA.5.1 subvariant had more prominent clinical symptoms such (fatigue, dyspnea, abdominal pain, headache, nausea) and higher viral loads in comparison with infection with BA.2.76 subvariant, which might be due to waning vaccine-induced immunity. Before diagnosis of COVID-19, a longer time after last vaccine was observed in the BA.5.1 group (364 vs. 281 days). Our results are consistent with the findings of prior research that demonstrate a decline in vaccine effectiveness over time regardless of Omicron subvariant type (11, 13). Further studies on this field are needed.

The present results showed that Azvudine was safe and effective for treatment of patients presenting with non-severe symptoms. This study reported 28.1% of the patients appeared general drug-related AEs, most of them were gastrointestinal symptoms, like nausea, diarrhea and upset stomach, which is similar to the total AEs rates (29.50%, 272/922) of Azvudine in a meta-analysis including six studies (22). Azvudine treatment effectively reduced the levels of inflammatory factors and increased the levels of anti-SARS-CoV-2 antibodies and immune cells compared with the control. These changes indicate improved immunity, which consequently shortened the virus shedding time and enhanced the recovery from SARS-CoV-2 infection. Recently, several research displayed that Azvudine significantly improved clinical symptoms and reduced the risk of severity in COVID-19 patients (22–25). Meanwhile, it also has good therapeutic effect in patients with compromised immune systems such as hemodialysis patients (26). Compared with other antiviral drugs, research on Azvudine fighting against SARS-CoV-2 was not enough. Further studies are needed to compare of Azvudine and other antiviral drugs, and application of Azvudine in specific populations.

The present study revealed that fever, Azvudine, lymphocyte counts, and anti-SARS-CoV-2 IgG titer were negatively correlated with NAN time. Among them, low levels of lymphocyte and anti-SARS-CoV-2 IgG were identified as independent risk factors for prolonged virus clearance time, consistent with findings from other studies on patients infected with the other Omicron variants (27) and Delta variants (28). Study by He (29) and Yin (30) reported that reduced level of lymphocytes is a potential indicator of the disease progression in patients infected with Omicron. As the COVID-19 becomes more severe, the lymphocyte count decreases, and the lung damage is exacerbated (31–35). Anti-SARS-CoV-2 IgG titer is negatively associated with the viral load. The levels of antibodies against SARS-CoV-2 decrease over time following vaccination, indicating the importance of routinely monitoring of the anti-SARS-CoV-2 antibodies kinetics and consistently administering COVID-19 vaccinations (36).

The present study has some limitations. First, the Omicron subvariant was defined based on the period of predominance, and not all cases were subjected to sequencing. Secondly, it should be considered that we only evaluated the safety and effectiveness of Azvudine in treating Omicron-infected patients in our study, further studies should focus on the comparing Azvudine with other antiviral treatments, and investigate the factors associated with non-response to Azvudine administration in multi-center or larger cohorts. Moreover, this study was conducted when COVID-19 was under control in China, patients infected with other Omicron substrains were not included, limiting the study’s external validity.

## 5 Conclusion

The present findings showed that patients infected with Omicron BA.5.1 were associated with frequent systemic symptoms and exhibited better profiles of laboratory parameters than patients infected with BA.2.76 subvariant. In addition, the results showed that Azvudine is an effective and safe drug for treating non-severe COVID-19 cases. Future studies could compare Azvudine with other antiviral treatments, and explore the factors associated with non-response to antiviral administration, in multi-center or larger cohort with different disease severity. Clinicians can use lymphocyte counts and anti-SARS-CoV-2 IgG titer to predict patients with a longer NAN duration and utilize dyspnea symptom to identify subjects with pneumonia. Our study linked clinical symptoms to different subvariant, treatment, and clinical outcomes, in patients infected Omicron. These findings offer valuable insights for healthcare providers and policymakers, aiding in the formulation of appropriate health strategies to combat future infections and enhance patient prognosis during the ongoing Omicron wave.

## Data Availability

The raw data supporting the conclusions of this article will be made available by the authors, without undue reservation.

## Footnotes

^1^ https://www.gov.cn/xinwen/2022-06/28/content_5698168.htm

## References

[1] Mistry P Barmania F Mellet J Peta K Strydom A Viljoen IM. SARS-CoV-2 variants, vaccines, and host immunity. Front Immunol. (2021) 12:809244. doi: 10.3389/fimmu.2021.809244 35046961 PMC8761766

[2] Suzuki R Yamasoba D Kimura I Wang L Kishimoto M Ito J. Attenuated fusogenicity and pathogenicity of SARS-CoV-2 omicron variant. Nature. (2022) 603:700–5. doi: 10.1038/s41586-022-04462-1, PMID: 35104835 PMC8942852

[3] Adjei S Hong K Molinari NM Bull-Otterson L Ajani UA Gundlapalli AV. Mortality risk among patients hospitalized primarily for COVID-19 during the omicron and Delta variant pandemic periods - United States, April 2020-June 2022. MMWR Morb Mortal Wkly Rep. (2022) 71:1182–9. doi: 10.15585/mmwr.mm7137a4, PMID: 36107788 PMC9484808

[4] Karyakarte RP Das R Dudhate S Agarasen J Pillai P Chandankhede PM. Clinical characteristics and outcomes of laboratory-confirmed SARS-CoV-2 cases infected with omicron subvariants and the XBB recombinant variant. Cureus. (2023) 15:e35261. doi: 10.7759/cureus.35261 36968876 PMC10035460

[5] Ikuse T Aizawa Y Yamanaka T Hasegawa S Hayashi T Kon M. Comparison of clinical characteristics of children infected with coronavirus disease 2019 between omicron variant BA.5 and BA.1/BA.2 in Japan. Pediatr Infect Dis J. (2023) 42:503–9. doi: 10.1097/INF.0000000000003894, PMID: 36916865

[6] Groza C Totschnig D Wenisch C Atamaniuk J Zoufaly A. A retrospective analysis of clinical features of patients hospitalized with SARS-CoV-2 omicron variants BA.1 and BA.2. Sci Rep. (2023) 13:7896. doi: 10.1038/s41598-023-34712-9 37193727 PMC10185952

[7] Gori Savellini G Anichini G Cusi MG. SARS-CoV-2 omicron sub-lineages differentially modulate interferon response in human lung epithelial cells. Virus Res. (2023) 332:199134. doi: 10.1016/j.virusres.2023.199134 37192725 PMC10191105

[8] Sun Y Wang M Lin W Dong W Xu J. Evolutionary analysis of omicron variant BF.7 and BA.5.2 pandemic in China. J Biosaf Biosecur. (2023) 5:14–20. doi: 10.1016/j.jobb.2023.01.002, PMID: 36718149 PMC9876032

[9] Huo D Yu T Shen Y Pan Y Li F Cui S. A comparison of clinical characteristics of infections with SARS-CoV-2 omicron subvariants BF.7.14 and BA.5.2.48 - China, October-December 2022. China CDC Wkly. (2023) 5:511–5. doi: 10.46234/ccdcw2023.096 37404291 PMC10316608

[10] Chen J Wang R Hozumi Y Liu G Qiu Y Wei X. Emerging dominant SARS-CoV-2 variants. J Chem Inf Model. (2023) 63:335–42. doi: 10.1021/acs.jcim.2c01352, PMID: 36577010 PMC9843632

[11] Lee JE Hwang M Kim YH Chung MJ Jeong WG Sim BH. Comparison of clinical outcomes and imaging features in hospitalized patients with SARS-CoV-2 omicron subvariants. Radiology. (2023) 308:e230653. doi: 10.1148/radiol.230653, PMID: 37462497

[12] Guo L Liu X Gu Y Jiang J Yang Z Lv Q. Distinct and relatively mild clinical characteristics of SARS-CoV-2 BA.5 infections against BA.2. Signal Transduct Target Ther. (2023) 8:171. doi: 10.1038/s41392-023-01443-2 37100801 PMC10132431

[13] Kang SW Park H Kim JY Lim SY Lee S Bae JY. Comparison of the clinical and virological characteristics of SARS-CoV-2 omicron BA.1/BA.2 and omicron BA.5 variants: a prospective cohort study. J Infect. (2023) 86:e148–51. doi: 10.1016/j.jinf.2023.01.015, PMID: 36669564 PMC9846898

[14] Lewnard JA Hong V Kim JS Shaw SF Lewin B Takhar H. Association of SARS-CoV-2 BA.4/BA.5 omicron lineages with immune escape and clinical outcome. Nat Commun. (2023) 14:1407. doi: 10.1038/s41467-023-37051-5 36918548 PMC10012300

[15] Yu B Chang J. Azvudine (FNC): a promising clinical candidate for COVID-19 treatment. Signal Transduct Target Ther. (2020) 5:236. doi: 10.1038/s41392-020-00351-z, PMID: 33040075 PMC7547293

[16] Yu B Chang J. The first Chinese oral anti-COVID-19 drug Azvudine launched. Innovation. (2022) 3:100321. doi: 10.1016/j.xinn.2022.100321, PMID: 36106026 PMC9461232

[17] Yang H Wang Z Wang C Zhang Y Han S An Z. Cost-effectiveness of Azvudine for high-risk outpatients with mild-to-moderate coronavirus disease 2019 in China. Clin Ther. (2024) 46:e1–5. doi: 10.1016/j.clinthera.2024.07.009, PMID: 39155175

[18] Chinese Center for Disease Control and Prevention. (2023). Epidemic Situation of Novel Coronavirus Infection in China. Available online at: https://wwwchinacdccn/jksj/xgbdyq/202411/t20241112_302584html

[19] National Health Commission and National Administration of Traditional Chinese Medicine. Novel Coronavirus Pneumonia Diagnosis and Treatment Plan (Trial Version 9). Chinese J Viral Dis. (2022) 12:161–9.

[20] Castro Dopico X Ols S Lore K Karlsson Hedestam GB. Immunity to SARS-CoV-2 induced by infection or vaccination. J Intern Med. (2022) 291:32–50. doi: 10.1111/joim.13372, PMID: 34352148 PMC8447342

[21] Fiolet T Kherabi Y MacDonald CJ Ghosn J Peiffer-Smadja N. Comparing COVID-19 vaccines for their characteristics, efficacy and effectiveness against SARS-CoV-2 and variants of concern: a narrative review. Clin Microbiol Infect. (2022) 28:202–21. doi: 10.1016/j.cmi.2021.10.005 34715347 PMC8548286

[22] Wang Y Xie H Wang L Fan J Zhang Y Pan S. Effectiveness of azvudine in reducing mortality of COVID-19 patients: a systematic review and meta-analysis. Virol J. (2024) 21:46. doi: 10.1186/s12985-024-02316-y, PMID: 38395970 PMC10893615

[23] de Souza SB Cabral PGA da Silva RM Arruda RF Cabral SPF de Assis A. Phase III, randomized, double-blind, placebo-controlled clinical study: a study on the safety and clinical efficacy of AZVUDINE in moderate COVID-19 patients. Front Med. (2023) 10:1215916. doi: 10.3389/fmed.2023.1215916, PMID: 37928473 PMC10620601

[24] Chen Z Tian F. Efficacy and safety of azvudine in patients with COVID-19: a systematic review and meta-analysis. Heliyon. (2023) 9:e20153. doi: 10.1016/j.heliyon.2023.e20153, PMID: 37809649 PMC10559905

[25] Yang H Wang Z Jiang C Zhang Y Zhang Y Xu M. Oral azvudine for mild-to-moderate COVID-19 in high risk, nonhospitalized adults: results of a real-world study. J Med Virol. (2023) 95:e28947. doi: 10.1002/jmv.28947, PMID: 37470209

[26] Shang S Fu B Geng Y Zhang J Zhang D Xiao F. Azvudine therapy of common COVID-19 in hemodialysis patients. J Med Virol. (2023) 95:e29007. doi: 10.1002/jmv.29007, PMID: 37522276

[27] Li H Zhu X Yu R Qian X Huang Y Chen X. The effects of vaccination on the disease severity and factors for viral clearance and hospitalization in omicron-infected patients: a retrospective observational cohort study from recent regional outbreaks in China. Front Cell Infect Microbiol. (2022) 12:988694. doi: 10.3389/fcimb.2022.988694, PMID: 36420118 PMC9677104

[28] Li H Lin H Chen X Li H Li H Lin S. Unvaccinated children are an important link in the transmission of SARS-CoV-2 Delta variant (B1.617.2): comparative clinical evidence from a recent community surge. Front Cell Infect Microbiol. (2022) 12:814782. doi: 10.3389/fcimb.2022.814782, PMID: 35350438 PMC8957884

[29] He S Fang Y Yang J Wang W. Association between immunity and viral shedding duration in non-severe SARS-CoV-2 omicron variant-infected patients. Front Public Health. (2022) 10:1032957. doi: 10.3389/fpubh.2022.1032957, PMID: 36620263 PMC9813739

[30] Yin R Lu Q Jiao JL Lin K Wang C Yuan L. Characteristics and related factors of viral nucleic acid negative conversion in children infected with omicron variant strain of SARS-CoV-2. Zhonghua Er Ke Za Zhi. (2022) 60:1307–11. doi: 10.3760/cma.j.cn112140-20220623-00582 36444435

[31] Jafarzadeh A Jafarzadeh S Nozari P Mokhtari P Nemati M. Lymphopenia an important immunological abnormality in patients with COVID-19: possible mechanisms. Scand J Immunol. (2021) 93:e12967. doi: 10.1111/sji.12967, PMID: 32875598

[32] Valyi-Nagy I Uher F Rakoczi E Szekanecz Z. Adaptive immunity to viruses: what did we learn from SARS-CoV-2 infection? Int J Mol Sci. (2022) 23:13951. doi: 10.3390/ijms232213951, PMID: 36430430 PMC9694482

[33] Khodeir MM Shabana HA Alkhamiss AS Rasheed Z Alsoghair M Alsagaby SA. Early prediction keys for COVID-19 cases progression: a meta-analysis. J Infect Public Health. (2021) 14:561–9. doi: 10.1016/j.jiph.2021.03.001, PMID: 33848885 PMC7934660

[34] Napoli C Benincasa G Criscuolo C Faenza M Liberato C Rusciano M. Immune reactivity during COVID-19: implications for treatment. Immunol Lett. (2021) 231:28–34. doi: 10.1016/j.imlet.2021.01.001, PMID: 33421440 PMC7787505

[35] Iwamura APD Tavares da Silva MR Hummelgen AL Soeiro Pereira PV Falcai A Grumach AS. Immunity and inflammatory biomarkers in COVID-19: a systematic review. Rev Med Virol. (2021) 31:e2199. doi: 10.1002/rmv.2199, PMID: 34260778

[36] Zhan H Gao H Liu Y Zhang X Li H Li X. Booster shot of inactivated SARS-CoV-2 vaccine induces potent immune responses in people living with HIV. J Med Virol. (2023) 95:e28428. doi: 10.1002/jmv.28428, PMID: 36571267 PMC9880704

