# Supplementary material for: Characteristics of patients with non-severe infections of different SARS-CoV-2 omicron subvariants in China
Source: Front Med (Lausanne). 2024 Dec 18;11:1511227. doi: 10.3389/fmed.2024.1511227 (PMC11688270; doi:10.3389/fmed.2024.1511227)
Supplement: Supplementary file 1 [file Table_1.DOCX]

**Supplementary materials**

**Supplementary table 1 The impact of vaccination status on clinical features of patients infected with Omicron BA.2.76 and/or BA.5.1**

| **Characteristics** | **BA.2.76** | | | **BA.5.1** | | |  |
| --- | --- | --- | --- | --- | --- | --- | --- |
|  | **Unvaccinated** | **Vaccinated** | **P value** | **Unvaccinated** | **Vaccinated** | **P value** | **P value *** |
| Number | 14 | 144 | / | 1 | 85 | / |  |
| **COVID-19 severity, n (%)** | | | | | | | |
| Asymptomatic | 8 (57.14%) | 55 (38.19%) | 0.252 | 0 (0%) | 25 (29.41%) | >0.999 | 0.171 |
| Mild | 4 (28.57%) | 74 (51.39%) | 0.160 | 1 (100%) | 52 (61.18%) | >0.999 | 0.116 |
| Moderate | 2 (14.29%) | 15 (10.42%) | 0.649 | 0 (0%) | 8 (9.41%) | >0.999 | 0.657 |
| **Symptoms, n (%）** | | | | | | | |
| Fever | 5 (35.71%) | 46 (31.94%) | 0.771 | 1 (100%) | 29 (34.12%) | 0.349 | 0.579 |
| Fatigue | 0 (0%) | 4 (2.78%) | >0.999 | 0 (0%) | 22 (25.88%) | >0.999 | 0.380 |
| Cough | 5 (35.71%) | 68 (47.22%) | 0.576 | 1 (100%) | 28 (32.94%) | 0.337 | >0.999 |
| Dyspnea | 0 (0%) | 0 (0%) | >0.999 | 0 (0%) | 5 (5.88%) | >0.999 | >0.999 |
| Expectoration | 4 (28.57%) | 29 (20.14%) | 0.493 | 0 (0%) | 19 (22.35%) | >0.999 | 0.532 |
| Sore throat/dry throat | 0 (0%) | 43 (29.86%) | **0.012** | 0 (0%) | 19 (22.35%) | >0.999 | **0.014** |
| Abdominal pain | 0 (0%) | 0 (0%) | >0.999 | 0 (0%) | 3 (3.53%) | >0.999 | >0.999 |
| diarrhea | 0 (0%) | 1 (0.69%) | >0.999 | 0 (0%) | 2 (2.35%) | >0.999 | >0.999 |
| Headache/dizziness | 0 (0%) | 7 (4.86%) | >0.999 | 0 (0%) | 11 (12.94%) | >0.999 | 0.611 |
| Nausea/vomit | 0 (0%) | 2 (1.39%) | >0.999 | 0 (0%) | 9 (10.59%) | >0.999 | >0.999 |
| Myalgia | 0 (0%) | 5 (3.47%) | >0.999 | 0 (0%) | 6 (7.06%) | >0.999 | >0.999 |

P value * indicated that the comparison of clinical symptoms between unvaccinated and vaccinated patient infected with Omicron subvariants (combining BA.2.76 and BA.5.1).

**Supplementary table 2 Baseline laboratory characteristics of patients infected with different Omicron subvariants**

| **Laboratory Characteristics** | **BA.2.76 group** | **BA.5.1 group** | **P value** |
| --- | --- | --- | --- |
| White blood cell (×10^9^/L) | 4.82 (3.86, 5.83) | 4.69 (3.57, 5.74) | 0.214 |
| Neutrophil (×10^9^/L) | 2.84 (2.11, 3.77) | 2.67 (1.78, 3.84) | 0.224 |
| Platelet (×10^9^/L) | 190 (158.75, 227.25) | 185 (153.5, 219.75) | 0.445 |
| D-Dimer (μg/L) | 320 (190, 462.29) | 380.85 (190, 478.14) | 0.811 |
| Alanine aminotransferase (U/L) | 18 (11.5, 26.5) | 18 (12, 30.75) | 0.594 |
| Aspartate amino transferase (U/L) | 21 (17, 26) | 22 (19, 29.75) | 0.085 |
| γ-glutamyltranspeptidase (U/L) | 18 (14, 33) | 19.5 (13.75, 34) | 0.812 |
| K^+^ (mmol/L) | 3.86 (3.59, 4.20) | 3.95 (3.63, 4.28) | 0.372 |
| Creatinine (μmoI/L) | 56 (48, 68) | 59 (49, 68) | 0.379 |
| Uric acid (μmol/L) | 301 (249, 403.5) | 325 (279, 389.5) | 0.099 |
| C-reactive protein (mg/mL) | 5.80 (2.80, 10.70) | 6.39 (3.60, 15.80) | 0.183 |
| Procalcitonin (ng/ml) | 0.05 (0.02 , 0.09) | 0.05 (0.02, 0.09) | 0.853 |

**Supplementary table 3. The comparison of Azvudine and control drug in the treatment of patients infected with different Omicron subvariant**

| **Characteristics** | **BA.2.76** | | | **BA.5.1** | | |
| --- | --- | --- | --- | --- | --- | --- |
|  | **Azvudine** | **Control** | **P value** | **Azvudine** | **Control** | **P value** |
| Number | 58 | 100 | / | 63 | 23 | / |
| Age (years) | 51.5 (38.75, 63.25) | 48 (33, 57.75) | 0.117 | 40 (36, 51) | 36 (31, 46) | 0.164 |
| Gender (Female/Male) | 30/28 | 40/60 | 0.153 | 31/32 | 12/11 | 0.808 |
| **COVID-19 severity, n (%)** | | | | | | |
| Asymptomatic | 12 (20.69%) | 51 (51%) | <0.001 | 19 (30.16%) | 6 (20.69%) | 0.794 |
| Mild | 31 (53.45%) | 47 (47%) | 0.510 | 37 (58.73%) | 16 (69.57%) | 0.456 |
| Moderate | 15 (25.86%) | 2 (2%) | <0.001 | 7 (11.11%) | 1 (4.35%) | 0.676 |
| **Vaccination status, n (%）** | | | | | | |
| Unvaccinated | 3 (5.17%) | 11 (11%) | 0.258 | 0 (0%) | 0 (0%) | 0.276 |
| Time after last vaccine (days) | 287 (239.8, 386) | 277 (241, 389) | 0.874 | 373 (321, 390) | 342.5 (263.3, 377.5) | 0.095 |
| Unvaccinated | 3 (5.17%) | 11 (11%) | 0.258 | 0 (0%) | 1 (4.35%) | 0.267 |
| 1 dose of vaccine | 0 (0%) | 1 (1%) | >0.999 | 1 (1.59%) | 0 (0%) | >0.999 |
| 2 doses of vaccine | 3 (5.17%) | 1 (1%) | 0.140 | 3 (4.76%) | 0 (0%) | 0.561 |
| 3 doses of vaccine | 52 (89.66%) | 87 (875) | 0.801 | 40 (63.49%) | 22 (95.65%) | 0.003 |

**Supplementary table 4 The difference of clinical and laboratory characteristic in Omicron infected patients with or without pneumonia**

|  | **Pneumonia**  **(n = 25)** | **No pneumonia**  **(n = 219)** | **P value** |
| --- | --- | --- | --- |
| **Clinical characteristic** | | | |
| Age (years) | 50(41,56) | 43(34,57) | 0.181 |
| Gender (F/M) | 12/13 | 119/100 | 0.602 |
| Subvariant  (BA.2.76/BA.5.1) | 17/8 | 141/78 | 0.720 |
| **Symptoms, n (%)** |  |  |  |
| Fever | 11(44%) | 70(31.96%) | 0.226 |
| Fatigue | 6(24%) | 20(9.13%) | **0.022** |
| Cough | 10(40%) | 92(42.01%) | 0.132 |
| Dyspnea | 3(12%) | 2(0.91%) | **0.008** |
| Expectoration | 7(28%) | 45(20.55%) | 0.862 |
| Sore throat and dry throat | 3(12%) | 59(26.94%) | 0.145 |
| Abdominal pain | 0(0%) | 3(1.37%) | >0.99 |
| diarrhea | 0(0%) | 3(1.37%) | >0.99 |
| Headache and dizziness | 2(8%) | 16(7.31%) | 0.693 |
| Nausea and vomit | 2(8%) | 9(4.11%) | 0.313 |
| Myalgia | 2(8%) | 9(4.11%) | 0.313 |
| **Vaccination status, n (%)** |  |  |  |
| 1^st^ dose of recombinant protein vaccine | 0(0%) | 1(0.46%) | >0.999 |
| 1^st^ dose of adenovirus vaccine | 0(0%) | 1(0.46%) | >0.999 |
| 2^nd^ dose of inactivated vaccine | 1(4%) | 5(2.28%) | 0.481 |
| 2^nd^ dose of recombinant protein vaccine | 0(0%) | 1(0.46%) | >0.999 |
| 3^rd^ dose of inactivated vaccine | 10(40%) | 112(51.14%) | 0.291 |
| 3^rd^ dose of recombinant protein vaccine | 11(44%) | 82(37.44%) | 0.523 |
| 3^rd^ dose of mixed vaccine | 1(4%) | 4(1.83%) | 0.420 |
| Unvaccinated | 2(8%) | 13(5.94%) | 0.657 |
| Time after last vaccine (days) | 343.5(276.8, 389.3) | 323(246, 385) | 0.328 |
| **Underlying disease, n (%)** | 10(40%) | 56(25.57%) | 0.107 |
| Hypertension | 6(24%) | 28(12.79%) | 0.125 |
| Diabetes | 2(8%) | 10(4.57%) | 0.354 |
| Cardiovascular disease | 1(4%) | 7(3.20%) | 0.585 |
| Cerebrovascular disease | 1(4%) | 4(1.83%) | 0.420 |
| Chronic lung disease | 1(4%) | 3(1.37%) | 0.363 |
| Chronic kidney disease | 0(0%) | 3(1.37%) | >0.999 |
| Chronic liver disease | 0(0%) | 5(2.28%) | >0.999 |
| Hematopathy | 1(4%) | 4(1.83%) | 0.420 |
| Malignant tumor | 3(12%) | 16(7.31%) | 0.424 |
| **Laboratory characteristic** | | | |
| CT-N | 18(15.23, 20.81) | 19.02(16.71, 23.04) | 0.167 |
| CT-O | 18.64(15.86, 20.37) | 19.11(16.73, 22.16) | 0.126 |
| Anti-SARS-CoV-2 IgG (AU/ml) | 5.99(0.74, 15.23) | 9.92(3.18, 35.66) | 0.101 |
| Anti-SARS-CoV-2 IgM (AU/ml) | 0.11(0.09, 0.25) | 0.11(0.02, 0.24) | 0.415 |
| White blood cell(×10^9^/L) | 4.71(3.45, 5.76) | 4.77(3.71, 5.77) | 0.565 |
| Neutrophil (×10^9^/L) | 3.14(2.12, 3.74) | 2.76(1.99, 3.83) | 0.567 |
| Lymphocytes (×10^9^/L) | 1.14(0.87, 2.11) | 1.6(1.13, 2.41) | 0.073 |
| Red blood cell (×10^12^/L) | 4.44(3.09, 4.6) | 4.4(3.85, 4.73) | 0.995 |
| Platelet (×10^9^/L) | 176(150, 223) | 187(159, 227) | 0.564 |
| D-dimer (μg/L) | 397(346.36, 500) | 307(190, 465) | **0.029** |
| Alanine aminotransferase (U/L) | 16(10, 24) | 18(12, 30) | 0.130 |
| Aspartate amino transferase (U/L) | 22(18, 26) | 21.5(17, 27) | 0.747 |
| γ-glutamyltranspeptidase (U/L) | 24(17, 37) | 18(13, 33) | 0.206 |
| K^+^ (mmol/L) | 3.71(3.42, 4.04) | 3.92(3.62, 4.22) | 0.097 |
| Creatinine (μmoI/L) | 58(55, 66) | 56(48, 68) | 0.454 |
| Uric acid (umol/L) | 322(268, 380) | 309(265, 403) | 0.826 |
| C-reactive protein (mg/mL) | 6.945(2.35, 10.78) | 5.91(2.9, 11.49) | 0.775 |
| Procalcitonin (ng/ml) | 0.07(0.035, 0.1) | 0.05(0.02, 0.09) | 0.214 |
| Interleukin-2 (pg/ml) | 2.5(2.5, 2.5) | 2.5(2.5, 2.5) | 0.153 |
| Interleukin-8 (pg/ml) | 5(5, 8.41) | 6.7(5, 63.69) | 0.194 |
| Tumor necrosis factor-α (pg/ml) | 3(3, 3.2) | 3(3, 3.39) | 0.782 |
| Interleukin-6 (pg/ml) | 3.875(1.33, 7) | 3.03(1.8, 6.30) | 0.910 |
| Interleukin-1β (pg/ml) | 3(3, 3) | 3(3, 3) | 0.924 |
| Interleukin-10 (pg/ml) | 3(3, 3.11) | 3(3, 3.06) | 0.723 |

Note: All laboratory index was detected before treatment.

Abbreviation: CT-N: cycle threshold of nucleocapsid protein, CT-O: cycle threshold open read frame.


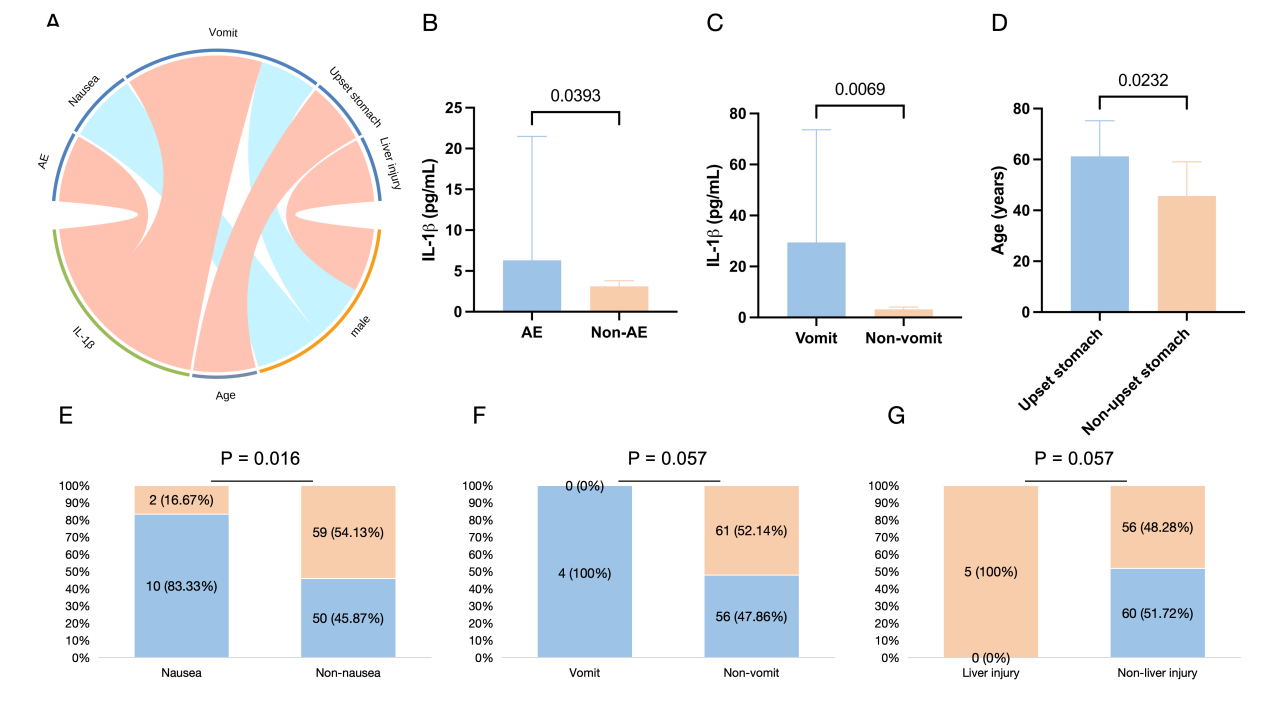


**Supplementary Figure 1. Factors associated with adverse events (AE) after Azvudine administration.** A. String plot showed that interleukin (IL) -1β levels before treatment, age, and gender were related to AE. The blue line represents the negative correlation and the orange represents the positive correlation. B-C. Patients with AE (B), or vomit (C) had higher levels of IL-1β than those without. D. Box plot demonstrated the difference of age in patients with or without upset stomach. E-G. AE of nausea (E) and vomit (F) were more often occurred in male patients, while liver injury (G) was the opposite. The blue and orange boxes represent the proportion of female and male, respectively.


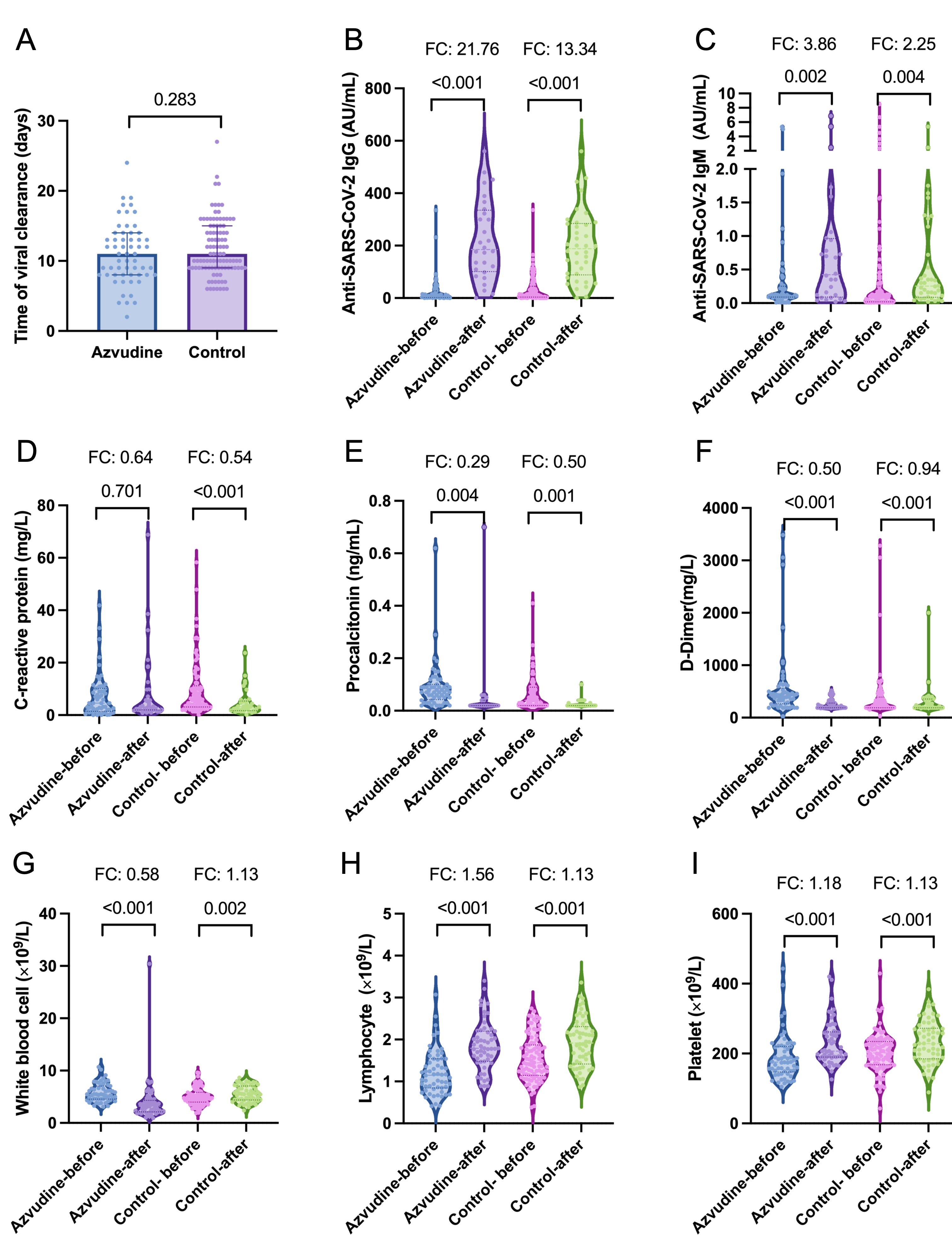


**Supplementary Figure 1. Azvudine improved the immune and inflamatory status of patients infected with BA.2.76.** A. The time of viral clearance in Azvudine and control groups were 11.07 days and 11.83 days, respectively. B-C. Azvudine treatment significantly enhanced the titers of anti-SARS-CoV-2 IgG (B) and IgM (C) compared to control treatment. D-F. The changes of C-reactive protein (D), procalcitonin (E), and D-dimer (F) in patients infected with BA.2.76 before and after azvudine and control treatment. G-H. After treatment, lymphocyte counts (H) and platelet (I) of patients receiving azvudine increased more than those in control group, except white blood cells (G).


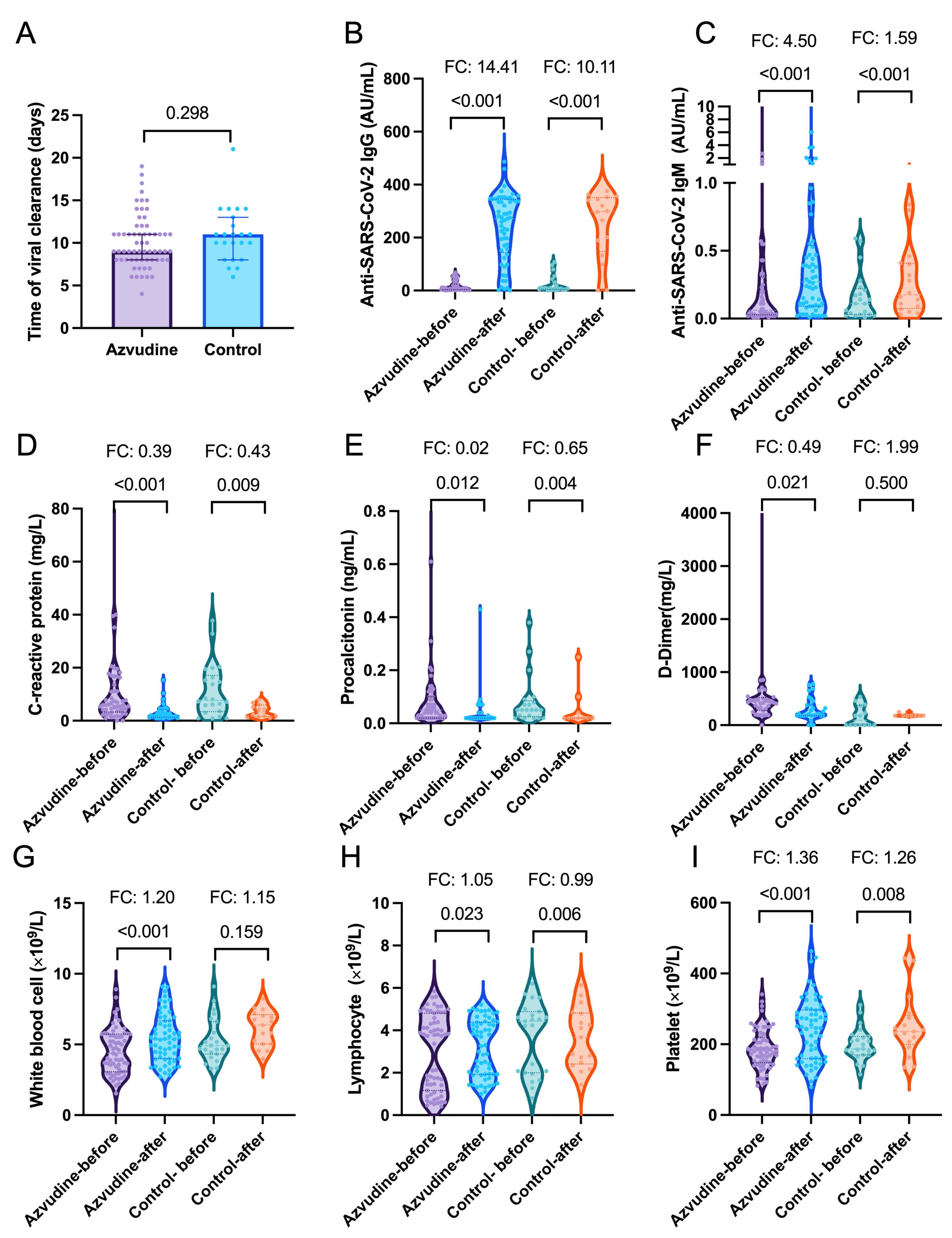


**Supplementary Figure 2. Azvudine improved the immune and inflamatory status of patients infected with BA.5.1.** A. The time of viral clearance was shorter in Azvudine group (10.14 days) than control group (10.91 days). B-C. Azvudine treatment significantly enhanced the titers of anti-SARS-CoV-2 IgG (B) and IgM (C) compared to control treatment. D-F. The reduction of C-reactive protein (D), procalcitonin (E), and D-dimer (F) in azvudine group was greater than that in control group. G-H. After treatment, immune cells including white blood cell counts (G), lymphocyte counts (H), and platelet (I) of patients receiving azvudine increased more than those in control group.
